# Multiple Near-Isogenic Lines Targeting a QTL Hotspot of Drought Tolerance Showed Contrasting Performance Under Post-anthesis Water Stress

**DOI:** 10.3389/fpls.2019.00271

**Published:** 2019-03-08

**Authors:** Md Sultan Mia, Hui Liu, Xingyi Wang, Guijun Yan

**Affiliations:** ^1^School of Agriculture and Environment, Faculty of Science, The University of Western Australia, Perth, WA, Australia; ^2^The UWA Institute of Agriculture, The University of Western Australia, Perth, WA, Australia; ^3^Plant Breeding Division, Bangladesh Agricultural Research Institute, Gazipur, Bangladesh

**Keywords:** drought tolerance, near-isogenic lines, quantitative trait loci, bread wheat, gene expression

## Abstract

The complex quantitative nature of drought-related traits is a major constraint to breed tolerant wheat varieties. Pairs of near-isogenic lines (NILs) with a common genetic background but differing in a particular locus could turn quantitative traits into a Mendelian factor facilitating our understanding of genotype and phenotype interactions. In this study, we report our fast track development and evaluation of NILs from C306 × Dharwar Dry targeting a wheat 4BS QTL hotspot in C306, which confers drought tolerance following the heterogeneous inbreed family (HIF) analysis coupled with immature embryo culture-based fast generation technique. Molecular marker screening and phenotyping for grain yield and related traits under post-anthesis water stress (WS) confirmed four isoline pairs, viz., qDSI.4B.1-2, qDSI.4B.1-3, qDSI.4B.1-6, and qDSI.4B.1-8. There were significant contrasts of responses between the NILs with C306 QTL (+NILs) and the NILs without C306 QTL (−NILs). Among the four confirmed NIL pairs, mean grain yield per plant of the +NILs and −NILs showed significant differences ranging from 9.61 to 10.81 and 6.30 to 7.56 g, respectively, under WS condition, whereas a similar grain yield was recorded between the +NILs and −NILs under well-watered condition. Isolines of +NIL and −NIL pairs showed similar chlorophyll content (SPAD), assimilation rate (A), and transpiration rate (Tr) at the beginning of the stress. However, the +NILs showed significantly higher SPAD (12%), A (66%), stomatal conductance (75%), and Tr (97%) than the −NILs at the seventh day of stress. Quantitative RT-PCR analysis targeting the MYB transcription factor gene *Triticum aestivum* MYB 82 (*TaMYB*82), within this genomic region which was retrieved from the wheat reference genome TGACv1, also revealed differential expression in +NILs and –NILs under stress. These results confirmed that the NILs can be invaluable resources for fine mapping of this QTL, and also for cloning and functional characterization of the gene(s) responsible for drought tolerance in wheat.

## Introduction

Global wheat production is often hampered by biotic and abiotic stresses like heat, drought, salinity, insects, and diseases. Among those stresses, drought is by far the most detrimental limiting the yield potential of wheat, particularly in rain-fed and limited irrigation environments (Akpinar et al., 2013; Budak et al., 2013). Effect of drought can be detected in various degrees during different growth periods including early establishment (Ayalew et al., 2015, 2016), pre-anthesis (Onyemaobi et al., 2017), and post-anthesis grain filling (Mia et al., 2017). Understanding the underlying mechanism of tolerance and identifying candidate genes and proteins will help to breed stress-resilient genotypes (Budak et al., 2015).

Although drought tolerance is a complex quantitative trait, a number of drought tolerance QTLs in wheat have been reported in various previous studies, most of which have been identified through grain yield and related component measurements under limited water conditions (Quarrie et al., 2006; Maccaferri et al., 2008; Mathews et al., 2008). However, large genomic intervals associated with those QTLs make them unsuitable for direct use in a breeding program. Characterizing those QTLs and identifying the causal genes, despite large genomic regions of interest, is quite a formidable task. This could be solved by developing near-isogenic lines (NILs) with different flanking markers of the respective QTL.

Near-isogenic lines have otherwise identical genetic backgrounds except at one or a few genetic loci and have been used intensively for detailed mapping and characterization of individual loci. Multiple pairs of isolines can offer a common genetic background to assess the phenotype conditioned by a genomic locus. Traditionally, NILs are developed through backcross introgression method (Kaeppler et al., 1993; Monforte and Tanksley, 2000; Babu et al., 2017). Alternatively, they can be generated following a selfing and selection scheme called heterogeneous inbreed family (HIF) analysis, which utilizes molecular markers linked to QTL of interest to identify heterozygous individuals, from which NILs can be extracted that are isogenic, but differ for certain genomic locations (Afanador et al., 1994; Tuinstra et al., 1997). Despite their usefulness for genetic and physiological studies of QTL, the considerable amount of time and effort needed to develop NILs have limited their use (Yan et al., 2017). However, a recently developed accelerated breeding technique for wheat called fast generation cycling system (FGCS) offers a suitable solution to this problem (Yan et al., 2017). Molecular marker assisted development of NILs following HIF analysis alone (Barrero et al., 2015), or coupled with FGCS, has been reported for some major QTLs in wheat (Ma et al., 2011; Wang et al., 2018) and barley (Habib et al., 2015). However, no such study has been reported for drought-tolerance related QTLs.

In this study, we utilize FGCS for fast track development of NILs targeting a QTL hotspot conferring drought tolerance following HIF analysis. Subsequently, those putative isolines were phenotyped for grain yield and related traits to characterize and confirm the NIL pairs. Several physiological parameters and relative gene expression were also evaluated for further confirmation and to explore the underlying mechanism of water stress (WS) tolerance during post-anthesis period in wheat.

## Materials and Methods

### Plant Materials

**FIGURE 1:**
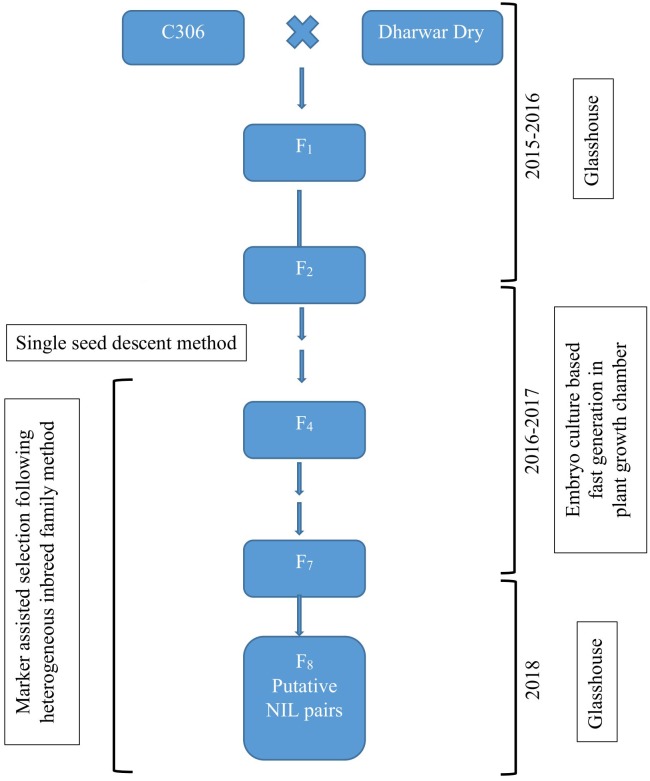
Schematic representation of the development of near-isogenic lines following heterogeneous inbreed family (HIF) method coupled with embryo culture-based fast generation technique.

Two bread wheat varieties of spring type growth habit, C306 and Dharwar Dry, were crossed to produce hybrids and subsequent segregating generations for this study. Several previous studies reported C306 (RGN/CSK3//2^∗^C591/3/C217/N14//C281) as a drought tolerant variety containing major-effect drought yield QTL (Aggarwal and Sinha, 1987; Sharma and Kaur, 2008; Kadam and Chuan, 2016). During crossing, C306 served as female parent, and Dharwar Dry was used as the male parent. NILs were developed from this cross targeting a QTL hotspot of 12 cM interval in C306 background by the HIF method (Tuinstra et al., 1997), coupled with immature embryo culture-based FGCS (Zheng et al., 2013; Yan et al., 2017). The procedure is summarized in Figure 1.

### Heterogeneous Inbreed Families (HIF) Method

Heterogeneous inbreed family analysis as described in Tuinstra et al. (1997) was followed for identifying NILs that differ for selected markers linked to a QTL of interest. In short, segregating generations from the biparental hybrids were advanced following single seed descent method till F_4_, where heterozygous plants were identified using the linked marker and selfed to produce seeds for next generation. For this study, marker gwm368 linked to the QTL *qDSI.4B.1* (Kadam et al., 2012) was used to identify heterozygous individuals for the targeted QTL in advancing generations. Six to eight plants derived from each of the heterozygous plants were used for the next round of selection, genotyped with the linked marker, and only a single heterozygous plant from each progeny line was selected and selfed. This process of selecting heterozygous individuals and selfing was repeated from F_4_ to F_7_. In F_7_, two isolines, homozygous but having different parental alleles, were isolated from the individual heterozygous plant progenies. These isolines served as putative NIL pairs for further phenotypic and physiological characterization in F_8_ (Figure 1).

### Embryo Culture-Based Fast Generation Technique

**FIGURE 2:**
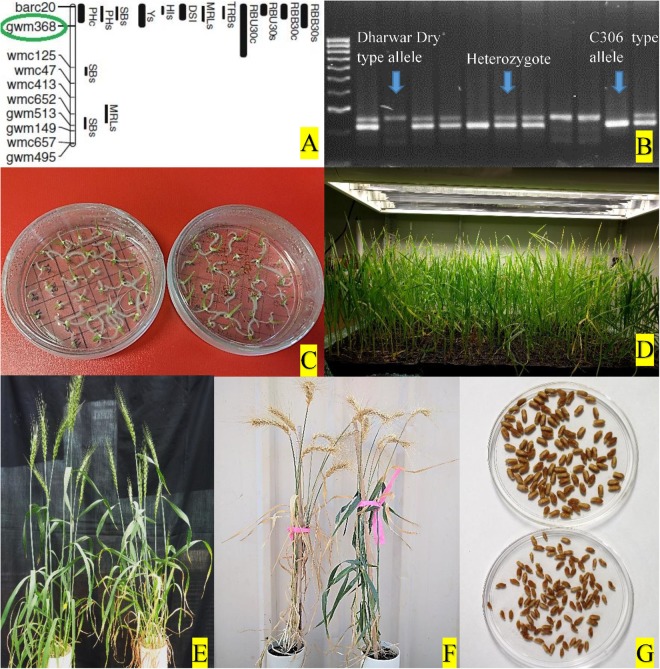
**(A)** QTL hotspot in wheat chromosome 4BS and the marker (circled in green) used for selection, adapted from Kadam et al. (2012). **(B)** Selection of different progeny types using molecular marker. **(C)** Culture of young embryos on petri-plates with sterile media. **(D)** Young seedlings from embryo culture growing in plant growth chamber. **(E)** NIL pair at anthesis in glasshouse. **(F)** NIL pair at physiological maturity. **(G)** Hundred seeds of a NIL pair.

For rapid generation advancement, an embryo culture-based FGCS as described in Yan et al. (2017) was followed from F_3_ to F_7_ (Figure 1, 2). In each generation, about 12–14 days after anthesis (DAA), young embryos were harvested from sterilized developing grains in aseptic condition and cultured on suggested medium. Cultured embryos in petri plates were kept in a specially designed plant growth chamber in the dark to germinate and were then transferred into a 22°C constant temperature room with 16 h light (fluorescent lamps) period for rooting. When the roots of the young seedlings were about 2.0 cm long, they were transferred into 30-well Kwikpot trays (Rite-Gro Kwikpots, GardenCity Plastics) containing growing media in plant growth chambers until the soft dough (Zadoks growth scale Z85) stage, when grains were ready for next cycle of embryo culture (Figure 2C,D).

### Genotyping of the Segregating Populations and NILs

Genomic DNA was isolated from leaf tissues collected from seedlings of two to three leaves stage following the CTAB method with necessary modifications (Dreisigacker et al., 2017). Genomic RNA contamination was removed from the extracted DNA by treating with RNaseA. The quality of the treated DNA was assessed by a NanoDrop 2000 spectrophotometer (ND-2000, Thermo Fisher Scientific, Inc., United States) and concentration was adjusted as per requirement. Integrity of the DNA was also checked by 1% agarose gel electrophoresis. Polymerase chain reaction (PCR) amplification was performed in an Eppendorf Mastercycler with 15 μl reaction volumes for each sample containing 50 ng template DNA, 200 nM of each primer, 1.5 μl 10 × PCR buffer, 2 mM MgCl_2_, 0.2 mM dNTPs, and 1 unit TaqDNA polymerase (Fisher Biotec) with initial denaturation at 94°C for 3 min, followed by 40 cycles of 94°C for 30 s, annealing at 58°C for 30 s, and 72°C for 30 s, with a final extension at 72°C for 5 min. PCR products were electrophoresed in 2.5% agarose gel, stained with ethidium bromide, and visualized with UV trans-illuminator. All plants were genotyped with the gwm 368 marker and grouped according to the genotypes “+ +,” “+−,” and “− −,” where “+” represents allele of C306 type and “-” represents allele of Dharwar dry type. The DNA profile from each marker was scored in each generation and only heterozygous plants (‘+’−) were selected for the next cycle, except for the last cycle (F_7_) when homozygous progeny (“+ +,” and “−−”) from each of those heterozygous plants were selected as NIL pairs (Figure 2).

### Phenotyping of the NILs (F_8_)

Near-isogenic lines were phenotyped following the procedure as described in Mia et al. (2017) in a temperature-controlled and naturally lit glasshouse at The University of Western Australia, Crawley, Western Australia (31°59′ S, 115°49′ E) in 50 cm × 9 cm cylindrical pots containing 2.5 kg of soil media (5:2:3 compost: peat: sand, pH∼6.0). The experiment was arranged in a completely randomized block design with three replicates. Field (pot) capacity of the soil media was initially measured in three free draining pots, each containing 2.5 kg of air-dry soil media, by inundating the pots with water and allowing them to drain for 48 h. Three random samples from each pot were taken, and their weights were measured using a balance before and after oven-drying to calculate per cent water content of the media at filed capacity using the following formula: %soil water content = $\frac{\mathrm{FW}-\mathrm{DW}}{\mathrm{DW}}$, where FW = fresh weight and DW = dry weight of the samples. Pots were watered and maintained at around 80% field capacity (FC) by weighing and manual watering on each alternate days until anthesis, Zadoks growth scale Z60 for cereals (Zadoks et al., 1974). At anthesis, two water treatments were implemented, where soil moisture in the well-watered (WW) treatment were maintained at about 80% FC by daily weighing and watering and continued until plants reached physiological maturity (Z91). In contrast, plants in the WS treatment were weighed but not watered for a period of 7 days from anthesis, with watering resuming on 7 DAA as per WW treatment and maintained until physiological maturity. At physiological maturity, plants were harvested manually and separated into shoots and roots. Grains were collected and dried at 35°C for 72 h and the rest of the shoots were oven-dried at 65°C until constant weight. Roots from individual pots were washed thoroughly and then oven-dried at 65°C until they reach a constant weight. Data on plant height at maturity, days to anthesis, days to maturity, spikelet numbers per spike, fertile tiller (having spikes with grain) per plant, shoot biomass, root biomass, harvest index, hundred grain weight, and grain yield per plant were recorded.

### Physiological Traits

The confirmed NIL pairs were also characterized for physiological traits under both stressed and stress-free conditions. Chlorophyll contents of the isolines were also measured at those times using a handheld portable chlorophyll meter (SPAD-502Plus; Konica Minolta, Osaka) just before treatment imposition and on 4 and 7 days after stress (DAS) treatment. Leaf gas exchange measurements (net photosynthesis rate, transpiration rate, and stomatal conductance) were also measured during those time points using a portable photosynthesis system (LI-6400, Li-COR Inc., Lincoln, NE, United States) with a LED light source on the leaf chamber. In the LICOR cuvette, CO_2_ concentration was set to 400 μmols^−1^ and LED light intensity was 1500 μmolm^−2^ s^−1^.

### RNA Extraction and Quantitative Reverse Transcriptase Polymerase Chain Reaction (qRT-PCR) Analysis

Flag leaf tissues from the NILs were snap frozen in liquid nitrogen and stored in −80°C for later use. For RNA extraction, leaf tissues from isolines with C306 background were bulked in one sample and those of Dharwar Dry background were bulked in another. There were three independent biological replicates, each with three technical replicates for both stressed and stress-free conditions. Total RNA was extracted using RNeasy^®^ Plant mini kit (Qiagen) with DNase digestion to eliminate genomic DNA contamination. Total RNA was assayed qualitatively and quantitatively by Nanodrop 2000, denatured gel electrophoresis, and LabChip^®^ GX Touch capillary electrophoresis (PerkinElmer). The cDNA was synthesized using a SensiFast cDNA Synthesis Kit (Bioline) following manufacturer’s protocol. Quantitative real-time PCR (qRT-PCR) was carried out with an ABI 7500 Fast system using SensiFast syber kit (Bioline). For gene expression analysis, *Triticum aestivum* MYB 82 (*TaMYB82*) gene, repeatedly found in this genomic region, was selected as target gene (Kadam et al., 2012; Liu et al., 2015). Primers (Forward 5′-TCGTCGGGTTCGTTCACATC-3′, Reverse 5′-GGTCGACGTGGAAAAGACCA-3′) were developed form the highly conserved exonic region of the MYB transcription factor gene, retrieved from chromosome 4BS of wheat reference genome TGACv1, using Geneious software version11.1.2 (Kearse et al., 2012). Wheat actin gene (Forward 5′-CTCCCTCACAACAACCGC-3′, Reverse 5′-TACCAGAACTTCCATACCAAC-3′) was used as endogenous control (Yu et al., 2017). Amplification was performed in a 20 μl final reaction mix containing 100 ng cDNA, 10 μl of 2X SensiFast SYBR Lo-ROX mix, and 0.8 μl of each primer (10 μM) with the following protocol: 95°C for 2 min (1 cycle), 95°C for 5 s, and 62°C for 30 s (40 cycle), melt curve analysis concluding with a 4°C hold. Relative gene expression was determined by the comparative Ct method (Livak and Schmittgen, 2001; Schmittgen and Livak, 2008).

### Statistical Analysis

Statistical analysis was performed using GenStat software, 17th edition (Payne et al., 2014). *t*-test was used to compare the difference between various means. Graphs were produced using statistical software R 3.5.1 with R package “ggplot2” and “gridExtra” (R Core Team, 2013; Wickham, 2016).

## Results

### Development of the Near-Isogenic Lines

Twenty-one F_1_ seeds were generated from the cross between C306 and Dharwar Dry. Hybridity of the F_1_ seeds was confirmed using the flanked marker gwm368. A total of 310 seeds from the best performing hybrid were grown from F_2_ to F_4_ following single seed descent method. 275 F_4_ plants were screened with the QTL flanking SSR marker gwm368 and 21 were found to be heterozygous. Six to eight seeds from each of those 21 plants were grown and screened with the marker to select only heterozygotes until F_7_ where two homozygous plants, one with + allele and another with – allele, from the same progeny lines were selected. At the end of F_7_, 14 putative isoline pairs were recovered and 10 pairs having similarity in flowering time and morphology were selected for phenotyping at F_8_.

### Putative Isolines Vary for Grain Yield and Grain Weight Under Post-anthesis Water Stress

Grain yield and grain weight of the 10 putative NIL pairs under WW and post-anthesis WS condition are given in Table 1. In general, post-anthesis WS caused significant reduction in both grain weight and grain yield, though there was sharp contrast in plants’ responses between the NIL with C306 background (termed as +NIL) and the corresponding NIL with Dharwar Dry background (−NIL) of the same pair. In the WW, the grain yield per plant and 100 grain weight of the NILs ranges from 10.56 to 15.52 and 4.45 to 5.60 g, respectively. By contrast, the grain yield per plant and 100 grain weight of the NILs ranges from 4.57 to 10.81 and 3.19 to 5.13 g, respectively, under stressed condition. On average, post-anthesis WS caused 42.57% reduction in grain yield and 12.51% in grain weight.

**Table 1:** Grain yield (g/plant) and 100 grain weight (g) of the developed NIL pairs.

NIL pairs	Grain yield (g/plant)				100 grain weight (g)			
	Well-watered	*p*	Water stressed	*p*	Well-watered	*p*	Water stressed	*p*
qDSI.4B.1-1(+)	12.48 ± 0.74	ns	7.30 ± 0.58	ns	5.04 ± 0.21	ns	4.33 ± 0.17	ns
qDSI.4B.1-1(−)	11.74 ± 0.68		6.65 ± 0.52		4.46 ± 0.43		5.00 ± 0.59	
**qDSI.4B.1-2(+)**	**13.85 ± 0.22**	**ns**	**9.71 ± 0.35**	**s**	**4.92 ± 0.30**	**ns**	**4.72 ± 0.30**	**s**
**qDSI.4B.1-2**(−)	**13.20 ± 0.39**		**6.30 ± 0.36**		**5.21 ± 0.22**		**3.91 ± 0.22**	
**qDSI.4B.1-3(+)**	**13.56 ± 0.95**	**ns**	**9. 61 ± 0.56**	**s**	**5.191 ± 0.21**	**ns**	**5.13 ± 0.21**	**s**
**qDSI.4B.1-3**(−)	**13.51 ± 0.70**		**6.49 ± 0.38**		**4.876 ± 0.27**		**3.72 ± 0.27**	
qDSI.4B.1-4(+)	12.09 ± 1.00	ns	7.35 ± 0.15	ns	4.91 ± 0.22	ns	5.13 ± 0.11	s
qDSI.4B.1-4(−)	11.69 ± 0.55		6.63 ± 0.34		5.02 ± 0.13		3.72 ± 0.17	
qDSI.4B.1-5(+)	11.80 ± 1.23	ns	6.58 ± 0.11	ns	5.12 ± 0.41	ns	4.40 ± 0.18	ns
qDSI.4B.1-5(−)	11.22 ± 0.80		5.94 ± 0.51		5.42 ± 0.10		4.83 ± 0.24	
**qDSI.4B.1-6(+)**	**15.52 ± 0.47**	**ns**	**10.81 ± 0.29**	**s**	**5.614 ± 0.13**	**ns**	**4.75 ± 0.13**	**s**
**qDSI.4B.1-6**(−)	**14.19 ± 1.07**		**7.06 ± 0.24**		**5.061 ± 0.24**		**3.72 ± 0.24**	
qDSI.4B.1-7(+)	11.25 ± 0.14	ns	6.15 ± 0.11	ns	4.96 ± 0.16	ns	4.94 ± 0.04	ns
qDSI.4B.1-7(−)	10.56 ± 1.09		5.59 ± 0.04		4.91 ± 0.12		4.30 ± 0.51	
**qDSI.4B.1-8(+)**	**15.31 ± 0.61**	**ns**	**10.71 ± 0.37**	**s**	**5.28 ± 0.18**	**ns**	**4.74 ± 0.18**	**s**
**qDSI.4B.1-8**(−)	**14.24 ± 0.20**		**7.56 ± 0.43**		**5.26 ± 0.27**		**3.65 ± 0.27**	
qDSI.4B.1-9(+)	12.84 ± 0.16	s	8.81 ± 0.15	s	4.45 ± 0.18	ns	4.12 ± 0.21	ns
qDSI.4B.1-9(−)	14.21 ± 0.66		5.78 ± 0.11		4.68 ± 0.16		4.57 ± 0.07	
qDSI.4B.1-10(+)	11.20 ± 0.08	s	4.47 ± 0.05	s	4.527 ± 0.13	s	3.19 ± 0.16	s
qDSI.4B.1-10(−)	14.60 ± 0.52		9.16 ± 0.16		4.84 ± 0.15		4.37 ± 0.14	

*^∗^ns = non-significant at *P* ≤ 0.05; s = significant at *P* ≤ 0.05; + = isoline with C306 background, − = isoline with Dharwar Dry background; pairs in bold are confirmed NILs. The significant difference was calculated using Student’s t-test.*

No significant difference was observed between the majority of the NILs of the WW condition. However, in the stress treatment, significant differences between the NILs were observed in most of the cases. We were particularly interested in the isoline pairs which showed similar responses under stressed-free condition but varied significantly under stressed condition. Out of 10 putative NILs pair, only four pairs, viz., qDSI.4B.1-2, qDSI.4B.1-3, qDSI.4B.1-6, and qDSI.4B.1-8 showed such significant difference between the isolines for both grain yield and grain weight and were considered as confirmed NIL pairs. For example, +NIL and −NIL of qDSI.4B.1-3 was recorded with mean grain yield per plant of 13.56 and 13.50 g, respectively, under WW condition, whereas in the stressed condition, mean grain yield per plant of −NIL was 6.49 g, about 32% less than that of the corresponding +NIL (9.61 g). Similarly, +NIL produced 38% higher grain weight than the corresponding −NIL (3.72 g) under stress.

Differences between the isoline pairs of qDSI.4B.1-4 and qDSI.4B.1-9 were significant only for either grain weight or grain yield and therefore were not considered as true NIL pairs. In most cases, NILs with C306 background out-yielded the corresponding NIL with Dharwar Dry background. However, isoline qDSI.4B.1-10(−), which has an allele from Dharwar Dry, outperformed the corresponding isoline with C306 allele under both stressed and stress-free conditions.

### Other Phenological Traits of the Confirmed NILs

**FIGURE 3:**
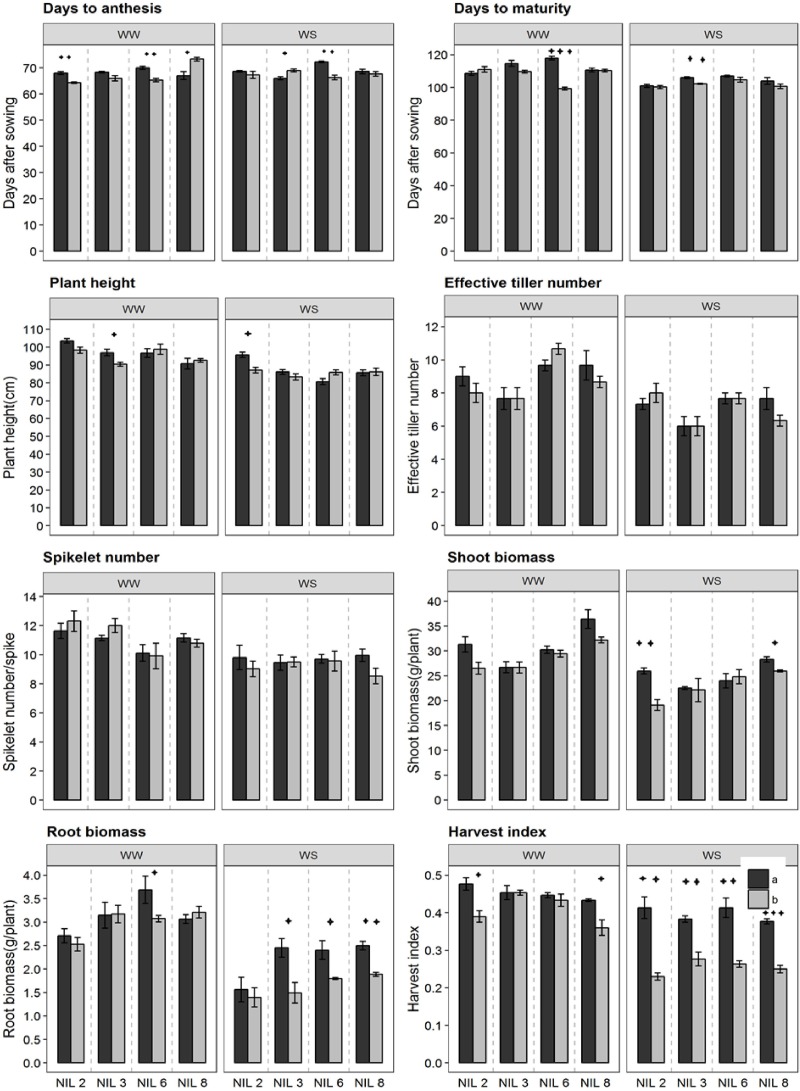
Different phenological traits of the confirmed NIL pairs. qDSI.4B1-2, qDSI.4B1-3, qDSI.4B1-6, and qDSI.4B1-8 are denoted as NIL 2, NIL 3, NIL 6, and NIL 8, respectively. a = NIL with C306 background (+NIL), b = NIL with Dharwar Dry background (–NIL), ^∗^ = significant at *P* ≤ 0.05, ^∗∗^ = significant at *P* ≤ 0.01, ^∗∗∗^ = significant at *P* ≤ 0.001.

Near-isogenic lines pairs commenced anthesis at around 68 days after sowing (Figure 3). However, under stressed treatment, they reach physiological maturity at around 103 DAS, about 1 week earlier than that under WW treatment (110 DAS). NIL pairs of qDSI.4B1-6 in the WW treatment and those of qDSI.4B1-3 in the stressed treatment differed significantly for days to maturity. Significant difference in plant height was observed only between the NIL pairs of qDSI.4B1-3 and qDSI.4B1-2 in WW treatment and stressed treatment, respectively. In both cases, +NIL was taller than the corresponding −NIL. NIL pairs did not differ significantly in terms of effective tiller number, and spikelet number across conditions.

In both stressed and non-stressed conditions, isoline qDSI.4B.1-2(+) and qDSI.4B.1-8(+), each of which has an allele form C306, showed higher shoot biomass than their corresponding isoline qDSI.4B.1-2(−) and qDSI.4B.1-8(−), respectively (Figure 3). By contrast, root biomass and harvest index were significantly lower in isolines with Dharwar Dry background in almost all of the four NIL pairs under stressed treatment. For example, under stressed condition, root biomass and harvest index of +NIL of qDSI.4B1-3 were about 40 and 27% lower, respectively, under stressed condition, than the corresponding −NIL, respectively.

### Physiological Traits of the Confirmed NILs

**FIGURE 4:**
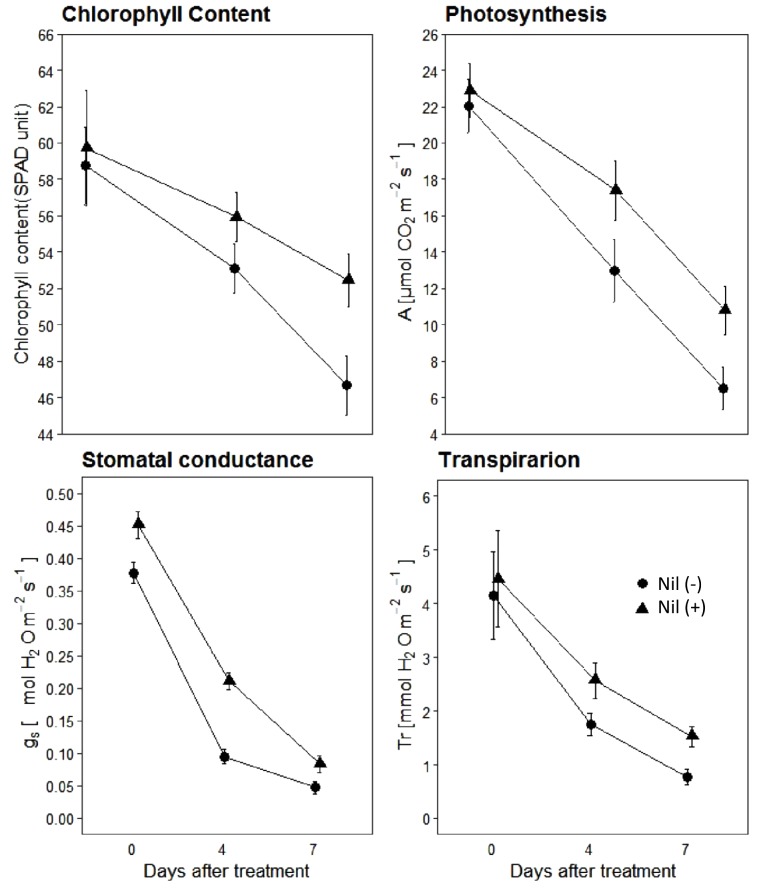
Chlorophyll content, photosynthesis (A; μmolm^−2^ s^−1^), stomatal conductance (gs; molm^−2^ s^−1^) and transpiration rate (T_r_; mmolm^−2^ s^−1^) of isolines of the confirmed NILs under post-anthesis water stress. Data points are mean ± SD (*n* = 12). Triangles and circles represent data points for NIL with C306 background (+NIL), and NIL with Dharwar Dry background (−NIL), respectively.

Results indicated that isolines of the NIL pairs responded variably in terms of physiological parameters when post-anthesis WS was applied (Figure 4). The magnitude of reduction varied between isolines of the NIL pairs with the increase of the stress period. Just prior to beginning of the stress period (0DAS), both the isolines had similar chlorophyll content, assimilation rate (A), stomatal conductance (g_s_), and transpiration (T_r_). However, with the progression of the stress period, both +NIL and −NIL showed significant decrease in A, g_s_, SPAD units, and T_r_. Overall, the +NIL maintained comparatively higher A, chlorophyll content, T_r_, and g_s_ than the –NIL during the stress period.

### Differential Expression of *TaMYB*82 Gene Between the Isolines

**FIGURE 5:**
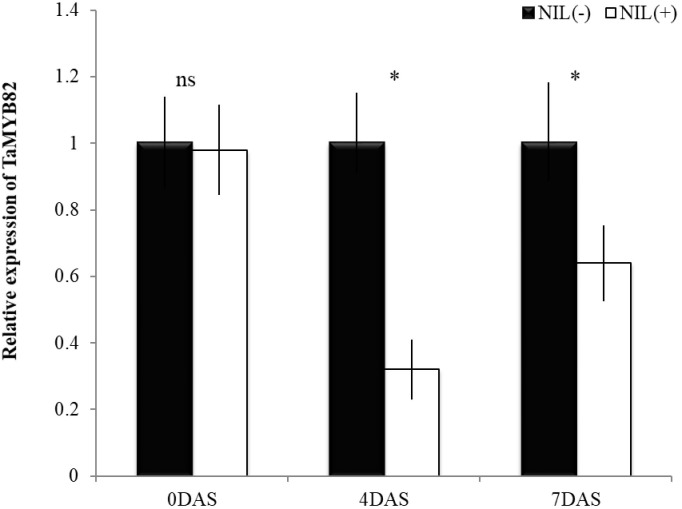
Relative expression of *TaMYB82* gene in +NIL and –NIL during stress period. Mean fold change in gene expression (2^−ΔΔC^T) of –NIL was set to 1 at each time point to determine relative expression in +NIL. Each value represents mean ± SD of three biological replicates with three technical replicates. ns = non-significant at *P* ≤ 0.01; ^∗^ = significant at *P* ≤ 0.01.

Relative expression of *TaMYB*82 gene was significantly different between +NIL and −NIL under post-anthesis WS at both time points (4 and 7 DAS; Figure 5). However, under WW condition (0 DAS), no significant difference was observed between the expression level of *TaMYB82* gene in +NIL and –NIL. At 4 DAS, there was about threefold difference in relative expression level of *TaMYB*82 gene between them. This difference was reduced but still significant at 7 DAS.

## Discussion

In this study, we reported the development of 10 putative pairs of NILs targeting a major locus for drought tolerance. Molecular screening and phenotyping of those NILs confirmed four true NILs which showed differential responses under well-watered and water-stressed conditions, as hypothesized. Among those, NILs having alleles from the C306 background showed improved performances in terms of grain yield and grain weight. This is because the nearby marker used for screening were tightly linked with the QTL identified for drought tolerance in parent C306, which may harbor some key genes responsible for grain yield and grain weight under post-anthesis stressed condition (Kadam et al., 2012). Several previous studies also reported that this genomic region is a rich hub for grain yield and related traits in spring wheat (Marza et al., 2006; Yang et al., 2007; Mathews et al., 2008; Pinto et al., 2010; Cabral et al., 2018).

Our study also reported that the NILs having allele from the C306 background are better performers in terms of physiological traits under post-anthesis WS. NILs with C306 background maintained comparatively higher photosynthesis, chlorophyll content, stomatal conductance, and transpiration rate compared to the corresponding NILs with Dharwar Dry background. One possible explanation of this might be the higher chlorophyll content and root biomass of the +NILs compared to the −NILs. Comparatively higher root biomass provides higher chances of accessing available soil moisture under stress (Wasson et al., 2012; Maeght et al., 2013). Kumar et al. (2012) reported several QTLs for photosynthesis and chlorophyll content in C306 background. Moreover, several previous studies indicated positive correlation of chlorophyll content and gas exchange parameters (Chen et al., 2015; Wang et al., 2016). This might be another possible explanation of the higher grain yield and grain weight in the tolerant NILs under stress.

Gene expression analysis of NILs revealed that *TaMYB*82 was markedly downregulated in the better performing NILs with C306 background when compared with the expression pattern of the NILs with Dharwar Dry background. This suggests that *TaMYB*82 acted as a negative regulator in response to the post-anthesis WS. The role of negative regulators of MYB transcription factors under drought has also been reported by Cui et al. (2013) and Jaradat et al. (2013). Furthermore, Zhao et al. (2017) indicated the role of *TaMYB*82 in an ABA-dependent signaling transduction pathway in response to abiotic stress. This supports the idea of involvement of MYB transcription factors in drought response mechanisms in wheat (Baldoni et al., 2015).

Although we identified four confirmed NIL pairs based on phenotyping and genotyping evaluation, there were six pairs of NILs, which were not in agreement with marker trait association. The possible explanation for this could be the recombination events between the targeted QTL and the nearby marker used for screening (Peleman et al., 2005). For example, in qDSI.4B.1-10, the isoline with Dharwar Dry background showed improved performance with respect to grain yield and grain weight compared to its counterpart, the NIL with the C306 background, under both WW and WS conditions. Moreover, despite having different genetic background, the isolines of NIL pairs qDSI.4B.1-1, qDSI.4B.1-5, and qDSI.4B.1-7 did not differ significantly in terms of grain yield and grain weight across conditions. Wang et al. (2018) also reported such phenomena in wheat during NIL production following HIF. Access to a higher resolution genetic map of this QTL locus saturated with more nearby markers would have solved this challenge. Yadav et al. (2011) described the utilization of multiple QTL-NILs for validation of drought tolerance QTL and how they were used for developing and mapping new gene-based markers in that genomic region. Therefore, fine mapping of this QTL using the confirmed NIL pairs reported in this study will provide an excellent opportunity to identify more closely linked markers, which will ensure higher selection efficiency (Gupta et al., 2017).

Transcriptomic analysis of multiple NILs enabled Barrero et al. (2015) to identify the candidate genes for a targeted QTL in wheat. Habib et al. (2018) also identified two key genes for a major dormancy QTL in wheat using similar multiple QTL-NILs RNA sequencing approach. Following these examples, next-generation transcriptome sequencing of the confirmed NILs under contrasting water regimes can be pursued as a future direction of the current study in order to identify the candidate genes in qDSI.4B.

Development of isolines for cereal crops requires substantial investment of time, regardless whether the traditional backcrossing or HIF method being followed. However, for this study, we utilized a rapid breeding technique which dramatically shortened the life cycles of segregating generations as reported in Zheng et al. (2013). Some drawbacks of this technique include dissecting of young embryo individually and culturing them *in vitro*, which requires considerable amount of effort and labor. Additionally, a sterile environment must be maintained during embryo culture. However, a recent discovery regarding shortening the life cycle of wheat by utilizing the longer photoperiod (22 h) with specialized LED lights might allow researchers to avoid the exploitation of immature embryo culture (Watson et al., 2018). Another limitation was the use of a single marker during marker-assisted selection. Between the two flanking markers (barc20 and gwm368) of the targeted QTL, only gwm368 was found to be polymorphic between the two parents. The other flanking maker barc20 was not polymorphic while the next closest neighboring marker (wmc125) were too far away (nearly 25 cM) from the targeted QTL. Hence, only gwm368 was used in the current study. Habib et al. (2015) and Wang et al. (2018) also successfully used one single marker to develop NILs in wheat and barley, respectively, following HIF method.

## Conclusion

In summary, the present study confirmed the importance of the 4BS QTL from the C306 background in post-anthesis drought tolerance. The confirmed NILs identified in this study are a valuable resource for future fine mapping of this QTL and for cloning and functional characterization of the gene(s) responsible for post-anthesis drought tolerance.

## Author Contributions

MM, HL, and GY conceived and designed the study. MM carried out the experimental procedures at plant growth chambers and greenhouse. MM and XW performed immature embryo culture and molecular marker assisted selection. MM collected all relevant data and performed analysis with occasional help from XW. MM prepared the manuscripts with inputs from XW, HL, and GY. All the authors reviewed the manuscript and approved the submission.

## Conflict of Interest Statement

The authors declare that the research was conducted in the absence of any commercial or financial relationships that could be construed as a potential conflict of interest.
